# Functionalization of gold-nanoparticles by the *Clostridium perfringens* enterotoxin C-terminus for tumor cell ablation using the gold nanoparticle-mediated laser perforation technique

**DOI:** 10.1038/s41598-018-33392-0

**Published:** 2018-10-08

**Authors:** Annegret Becker, Miriam Leskau, Barbara L. Schlingmann-Molina, Susanne C. Hohmeier, Suhayla Alnajjar, Hugo Murua Escobar, Anaclet Ngezahayo

**Affiliations:** 1 Institute of Cell Biology and Biophysics, Department of Biophysics and Cell Physiology, Leibniz University Hannover, Hannover, Germany; 2 Meissa Vaccines, South San Francisco, California, USA; 3 Small Animal Clinic, University of Veterinary Medicine Hannover, Hannover, Germany; 4 Division of Medicine, Haematology, Oncology and Palliative Medicine, University of Rostock, Rostock, Germany; 5 Center of System Neuroscience, University of Veterinary Medicine Hannover, Hannover, Germany

## Abstract

A recombinant produced C-terminus of the *C. perfringens* enterotoxin (C-CPE) was conjugated to gold nanoparticles (AuNPs) to produce a C-CPE-AuNP complex (C-CPE-AuNP). By binding to claudins, the C- CPE should allow to target the AuNPs onto the claudin expressing tumor cells for a subsequent cell killing by application of the gold nanoparticle-mediated laser perforation (GNOME-LP) technique. Using qPCR and immunocytochemistry, we identified the human Caco-2, MCF-7 and OE-33 as well as the canine TiHoDMglCarc1305 as tumor cells expressing claudin-3, -4 and -7. Transepithelial electrical resistance (TEER) measurements of Caco-2 cell monolayer showed that the recombinant C-CPE bound to the claudins. GNOME-LP at a laser fluence of 60 mJ/cm^2^ and a scanning speed of 0.5 cm/s specifically eliminated more than 75% of claudin expressing human and canine cells treated with C-CPE-AuNP. The same laser fluence did not affect the cells when non-functionalized AuNPs were used. Furthermore, most of the claudin non-expressing cells treated with C-CPE-AuNP were not killed by GNOME-LP. Additionally, application of C-CPE-AuNP to spheroids formed by MCF-7 and OE-33 cells grown in Matrigel reduced spheroid area. The results demonstrate that specific ablation of claudin expressing tumor cells is efficiently increased by activated C-CPE functionalized AuNPs using optical methods.

## Introduction

Despite advances in diagnostic and treatment, cancer is still a leading cause of death worldwide. Therefore, the development of new tools to tackle neoplastic and malignant cells while causing minimal harm to non-neoplastic cells is still an ongoing research goal addressed by different methodical approaches. In this context, analysis of tumor specific molecules that can be specifically targeted is a promising strategy^1^.

Among different tumor cell markers, the epidermal growth factor 2 receptor HER2 has attracted the research community. In about 25% of breast cancer diagnosed patients, HER2 is amplified. Because of the aggressive nature of HER2^+^ breast cancers, the amplification of HER2 correlates with poor prognosis^2,3^. Consequently, the use of HER2 antibody (Trastuzumab) was proposed as part of a new class of drugs. Although, treatment of HER2^+^ metastatic breast cancer revealed beneficial effects^4,5^, several patients developed a therapeutic resistance^2,6^. Other molecules targeting the EGF signaling system such as Lapatinib, a small molecule that inhibits tyrosine kinase, have been developed. However, like in the case of Trastuzumab, resistance to this molecule was observed^7^.

New approaches are oriented towards using gold nanoparticles mediated tumor cell killing as a new and minimally invasive method to eliminate malignant tumor cells^8^. For this, gold nanoparticles are applied to tumor cells. After adhesion onto the cells, the gold nanoparticles are activated by application of a laser beam. The interaction between the laser and the gold nanoparticles induces localized surface plasmon resonance (LSR) and heat generation, which irreversible perforate the cells resulting in cell death^9^. The efficiency of the method was shown *in vitro* and even in animal models. Studies showed that gold nanoparticles applied intravenously to animals, harboring a human tumor xenograft composed of SK-BR-3 cells, allowed a complete elimination of the tumor by an optical activation of the gold nanoparticles^10–12^. The challenge of this approach is to achieve a specific targeting of gold nanoparticles onto the cancer cells. In this context, the functionalization of gold nanoparticles with biological molecules recognizing target molecules specifically expressed in the membrane of tumor cells seems to be a promising option. Consequently, it was shown that anti-HER2 antibody functionalized gold nanoparticles bound six times better to tumor cells than non-functionalized gold nanoparticles^13^. Similarly, the usage of an antibody against transferrin receptor promoted the binding of gold nanoparticles on Neuro2A tumor cells which upregulate their expression of transferrin receptor^14^.

The aim of the present report was to analyze whether the C-terminus of the *C. perfringens* enterotoxin (C-CPE) could be used for a functionalization of gold nanoparticles in order to specifically address and kill tumor cells. The use of *C. perfringens* enterotoxin (CPE) to target tumor cells raised after it was observed that the development of many tumor types correlated with a dysregulated expression of claudin-3 -4 or -7^15–17^. In breast, esophagus and colon tumors these claudins are often upregulated which is frequently associated with poor survival of the patients^18–20^. The elevated expression of claudin-3, -4 and -7 in tumor development is particularly interesting since they are natural receptors for the CPE^21–25^. Accordingly, several studies using cell cultures and animal models showed that CPE was able to destroy cancer cells. It was shown, for example that CPE efficiently killed cancer cells derived from chemotherapy-resistant human ovarian cancer and implanted in an animal model^26^. Similarly, CPE destroyed human breast tumor xenografts when directly applied to the tumor. Unfortunately, intraperitoneal administration of the CPE to the animal in order to destroy the tumor was lethal to the animals although the dose were the same as those applied directly to the tumor^27^. The data strongly indicates that a systemic application of the complete CPE as therapeutic agent might cause deleterious side effects.

Combination of genetic, biochemical, and structural biologic methods to decipher the structure/function relationship of the 319 amino acid residues CPE polypeptide revealed that CPE is a three-domain protein, characteristic of several other pore forming toxins^28^. The C-terminus of the CPE (C-CPE; D194-F319) serves as the binding domain to claudin-3, -4 and -7^29–31^. Due to the C-CPE binding to a subset of claudins, C-CPE modulates the tight junctions and thus the barrier function of epithelial tissues without triggering cell death. Therefore, a functionalization of an active cell destroying component (e.g. nanoparticles) by C-CPE is thought to represent a promising valid approach in tumor therapy. As proof of principle, we propose to use a combination of the GNOME-LP photonic technique and C-CPE functionalized AuNPs (C-CPE-AuNP) to specifically kill claudin expressing tumor cells. The GNOME-LP technology has been used as a rapid method for a transient cell permeabilization allowing an uptake of molecules, like dyes or siRNA into cells^32–35^. The reversible cell permeabilization was achieved by heat generated through the interaction of a femtosecond laser pulse and gold nanoparticles randomly adherent on the cells. In these experiments, energy density of the applied laser beam was adjusted to achieve a transient cell permeabilization^32,35,36^, followed by closing of the cell membrane. Using C-CPE-AuNP, our hypothesis is that the AuNP should specifically bind claudin expressing cells and an adjusted laser energy should activate the AuNP to irreversible permeabilize the cells, inducing thereby their death.

This report demonstrates that a recombinant produced C-CPE construct can be used to functionalize gold nanoparticles (AuNP) in order to elicit tumor cell death by activation of the AuNPs using a laser beam in a photonic technique known as gold nanoparticle-mediated laser perforation (GNOME-LP). Implementation of functionalized gold nanoparticles was done by fusion the N-terminal domain of C-CPE with the Strep-Tag II. The Strep-Tag is a classical octo-amino acid peptide (W-S-H-P-Q-F-E-K) allowing a simple protein purification using affinity chromatography^37^. Importantly, the Strep-Tag warrants protein coupling to diverse materials such as chromophores or nanomaterials conjugated with the Strep-Tag ligand Strep-Tactin. Accordingly, our results show that the C-CPE could be used to functionalize Strep-Tactin conjugated chromophores for a claudin visualization at cell membranes, or Strep-Tactin conjugated gold nanoparticles for efficiently killing of claudin (claudin-3, -4 and -7) expressing tumor cells using the GNOME-LP technique. This report opens the possibility for a development of GNOME-LP technique with C-CPE functionalized gold nanoparticles for tumor therapy.

## Results

**Figure 1:**
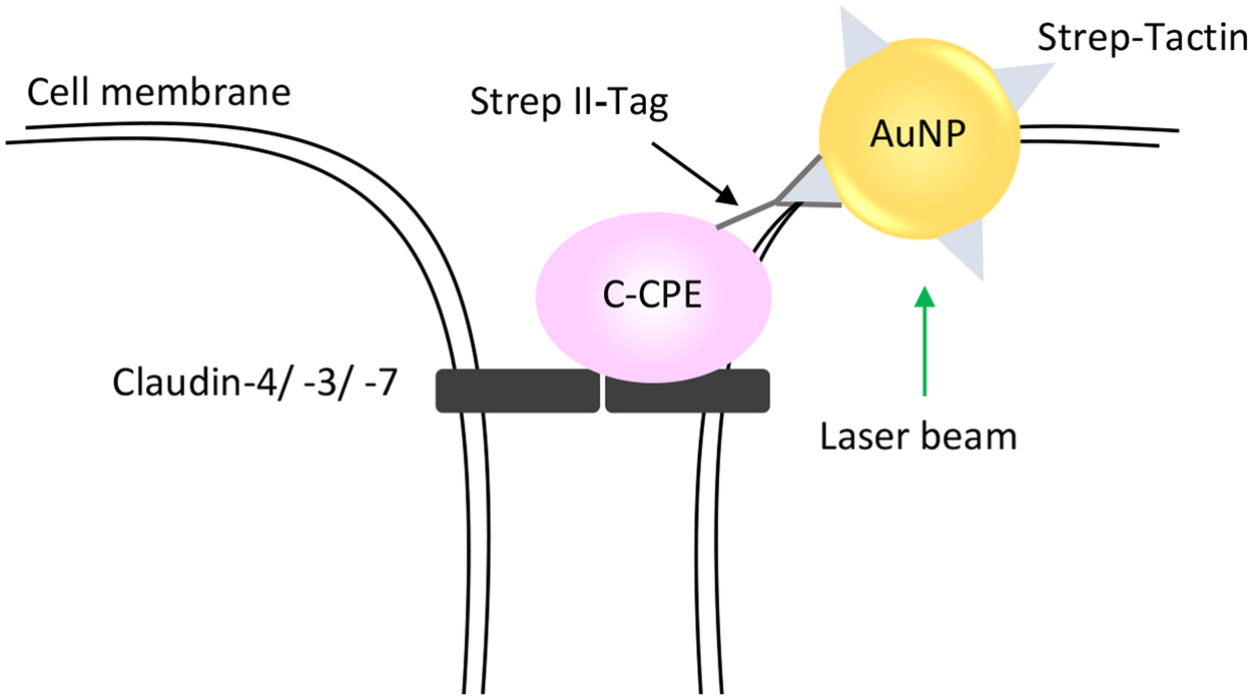
Schema depicting of the C-CPE-AuNP complex binding to claudin-3, -4 and -7 expressing tumor cell exposed to a laser beam.

The goal of our study was to test whether the C-CPE-AuNP complex composed of gold nanoparticles (AuNP) coupled to a recombinant produced C-terminus of *C. perfringens* enterotoxin (C-CPE) could be used for an efficient and specific killing of claudin expressing tumor cells (Fig. 1). Since, claudin-3, -4 or -7, the CPE receptors, can be upregulated in tumor cells, the use of C-CPE-AuNP could be a promising technique for a specific cancer therapy.

### Identification of tumor cells expressing claudin-3, -4 or -7

**Figure 2:**
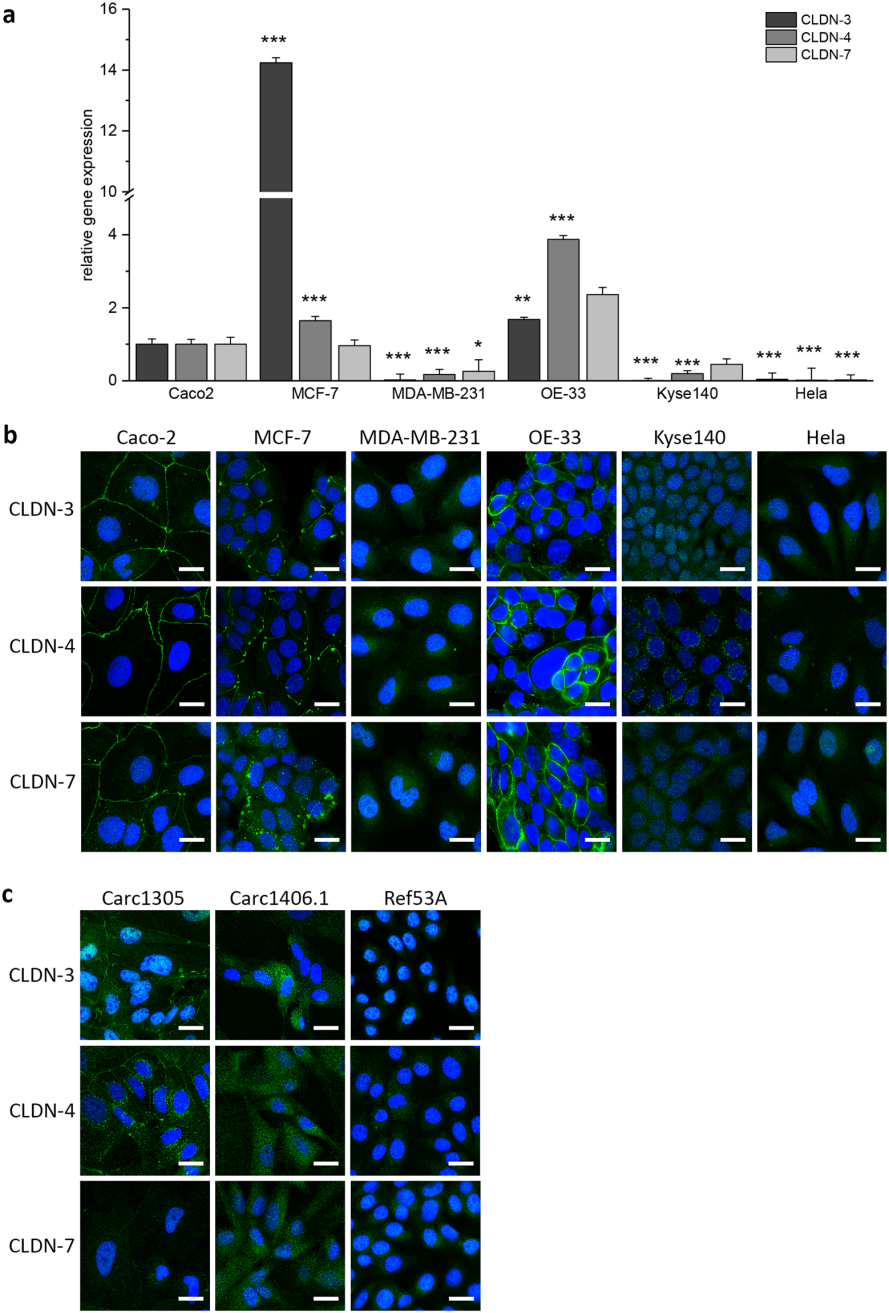
Claudin gene expression in different tumor cells (**a**) Quantification of claudin (CLDN) gene expression with qRT-PCR. Heterogeneous claudin-3, -4, -7 gene expression in MCF-7, MDA-MB-231, OE-33, Kyse140 and Hela cells. The graphs represent the mean ± SEM gene expression level of CLDN-3, -4 and -7 relative to GAPDH. The results were analyzed with Student’s t test. *Significant difference to the control reference: *P < 0.05, **P < 0.01 and **P < 0.001. (**b**,**c**) Immunostaining of claudins in human (**b**) and canine (**c**) tumor cells. Expression pattern of CLDN-3, -4 and -7 (green) was variable between tumor cells. In Caco-2, MCF-7 and OE-33 cells CLDN-3, -4 and -7 (green) are localized at cell-cell junctions. Signals of CLDN expression were absent in MDA-MB-231, Kyse140 and Hela cells. Canine cell lines Carc1305, express CLDN -3 and -4 but not CLDN-7 at cell-cell junctions whereas Carc1406.1 and Ref53A cells lack CLDN expression. Nuclei are stained with DAPI. Scale bar, 20 µm.

As human tumor cells, the colon adenocarcinoma cell line Caco-2, the cervix adenocarcinoma cell line Hela, the mammary gland adenocarcinoma cell lines MCF-7 and MDA-MB-231 as well as the esophageal squamous carcinoma cell line Kyse-140 and the esophageal adenocarcinoma cell line OE-33 were used. Real-time PCR with GAPDH and ACTB as housekeeping genes was performed for a quantitative comparison of the claudin expression between the different cells (Fig. 2a). Since, gene expression for GAPDH and ACTB were similar, data for ACTB are not shown. Caco-2 cells are known to express claudins, which allow the Caco-2 cells to form a very tight barrier as attested by measurements of the transepithelial electrical resistance (TEER)^38^. Correspondingly, the real-time PCR results for claudin-3, -4 and -7 detected in the different cells were normalized to the respective claudin expression found in Caco-2 cells. As shown in Fig. 2a, human cells used in this study can be classified in a group of high-expression of claudin comprising Caco-2, MCF-7, and the OE-33 versus a group of claudin low-expression cells comprising MDA-MB-231, Kyse140 as well as Hela. At the protein level, immunocytochemistry showed presence of claudin-3, -4 and -7 at cell-cell junctions in Caco-2, MCF-7 and OE-33 cells (Fig. 2b) which was expected from the PCR data for these claudin expressing cells. In Hela, MDA-MB-231, Kyse140 cells, the claudins were not detected by the immunostaining (Fig. 2b) suggesting to consider these cells as claudin non-expressing cells.

In previous works, expression of claudin-3, -4 and -7 was studied in cells derived from canine mammary tissues. Real-time PCR analysis showed a high expression of the claudins in the TiHoDMglCarc1305 cells, while TiHoDMglCarc1406.1 and TiHoDMglRef53A cells showed a very low expression of claudin-3, -4 and -7^39,40^. Similarly, for the human cells, immunocytochemistry experiments found claudin-3 and -4 at cell-cell junctions of TiHoDMglCarc1305 cells, while none of these claudins were detected in TiHoDMglCarc1406.1 and TiHoDMglRef53A cells (Fig. 2c). Taken together, the data identified the human Caco-2, MCF-7 and OE-33 cells as well as the canine TiHoDMglCarc1305 cells, expressing claudin-3, -4 and -7, as good candidates to test whether claudin expressing tumor cells can be specifically targeted using GNOME-LP technique in combination with the C-CPE-AuNP complex.

### The functionality of the recombinant C-CPE

**Figure 3:**
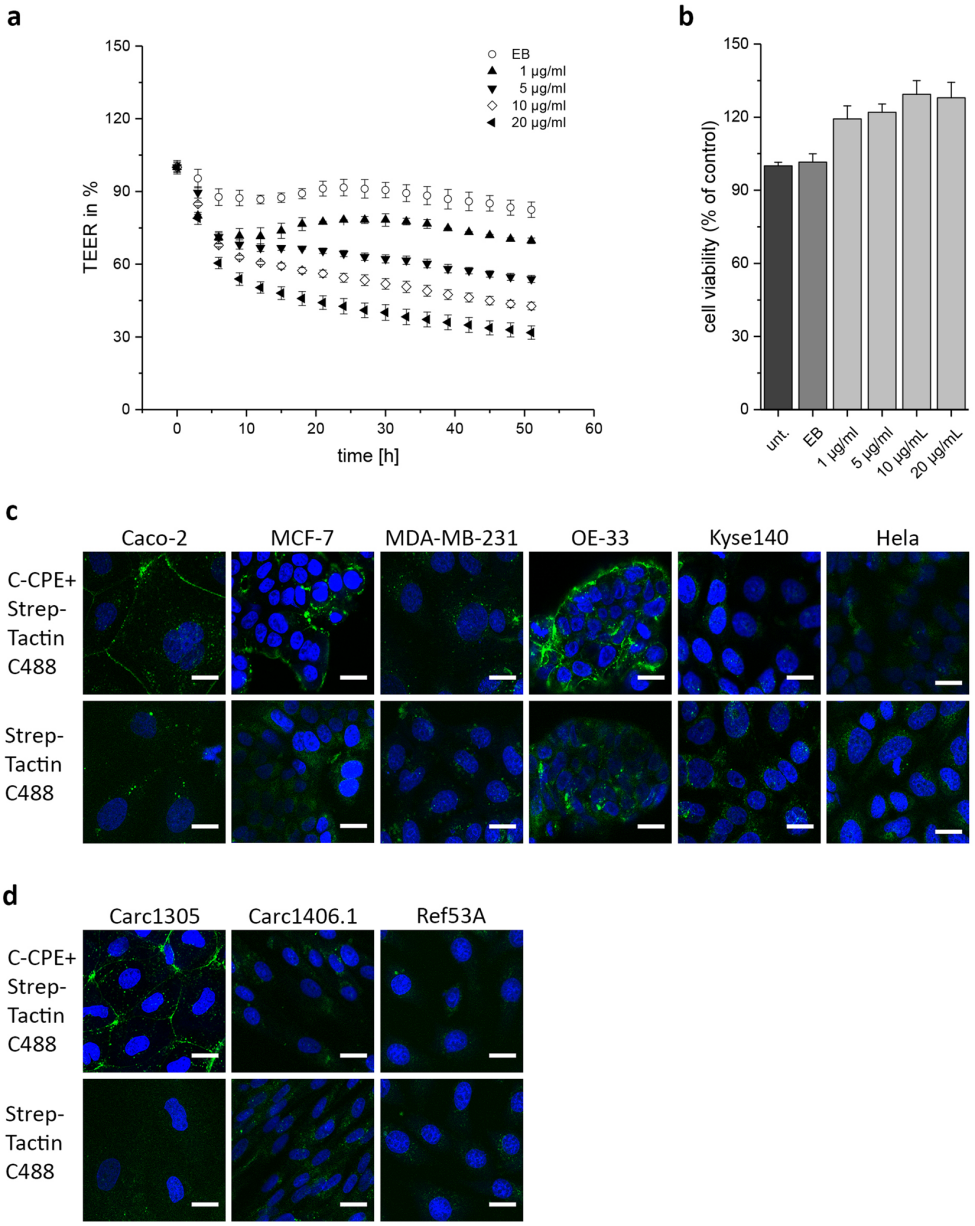
Functionality of the recombinant C-CPE. (**a**) TEER measurement of Caco-2 cell monolayer treated with different C-CPE concentrations. C-CPE reduced TEER of Caco-2 cells over time in a dose dependent manner. Elution buffer (EB) of C-CPE did not affect TEER. Graphs represent the mean ± SEM of TEER relative to untreated cells as control reference. (**b**) Cell viability analyzed with MTT cytotoxicity assay after 24 h C-CPE treatment. No effect of C-CPE application on cell viability. Graphs represent the mean ± SEM of cell viability relative to untreated cells as control reference. (**c**,**d**) Specific binding of C-CPE-Strep-Tactin Chromeo 488 complex (C-CPE + Strep-Tactin C488) on claudin (CLDN) expressing cells. C-CPE binding at cell-cell junctions of Caco-2, MCF-7, OE-33 and Carc1305 cells (green). No binding of C-CPE on MDA-MB-231, OE-33, Kyse140, Hela, Carc1406.1 and Ref53A cells. Application of 5 µg/ml C-CPE conjugated to Strep-Tactin Chromeo 488 for 3 h. Nuclei are stained with Hoechst. Scale bar, 20 µm.

To investigate whether our recombinant produced C-CPE bound to claudins, we performed two types of experiments. First, we analyzed whether the recombinant produced C-CPE was efficient in reduction of TEER of Caco-2 cell monolayer cultivated on the membrane of transwell inserts. TEER measurements were performed for post-confluent monolayer Caco-2 with a TEER of 800–1000 Ω · cm². In a time- and dose-dependent manner, 1–20 µg/ml C-CPE constantly reduced the TEER by 30–60% (Fig. 3a). Additionally, the non-cytotoxicity of C-CPE^41^ was confirmed with the MTT viability assay (Fig. 3b). Secondly, the C-CPE was coupled to Strep-Tactin Chromeo 488 and applied to the different tumor cells for 3 h. The C-CPE-Chromeo 488 complex marked the boundaries of claudin expressing human Caco-2, MCF-7, OE-33 as well as canine TiHoDMglCarc1305 cells but not the claudin non-expressing Hela, MDA-MB-231 and Kyse140 cells or TiHoDMglCarc1406.1 and TiHoDMglRef513A cells (Fig. 3c,d). In summary, the data show that the recombinant produced C-CPE construct bound specifically to claudins at cell-cell junctions between adjacent cells and thus dose-dependently disturbed the barrier function of tight junctions without affecting the viability of the cells.

### Application of the C-CPE-AuNP complex for an optic induced killing of claudin expressing tumor cells

**Figure 4:**
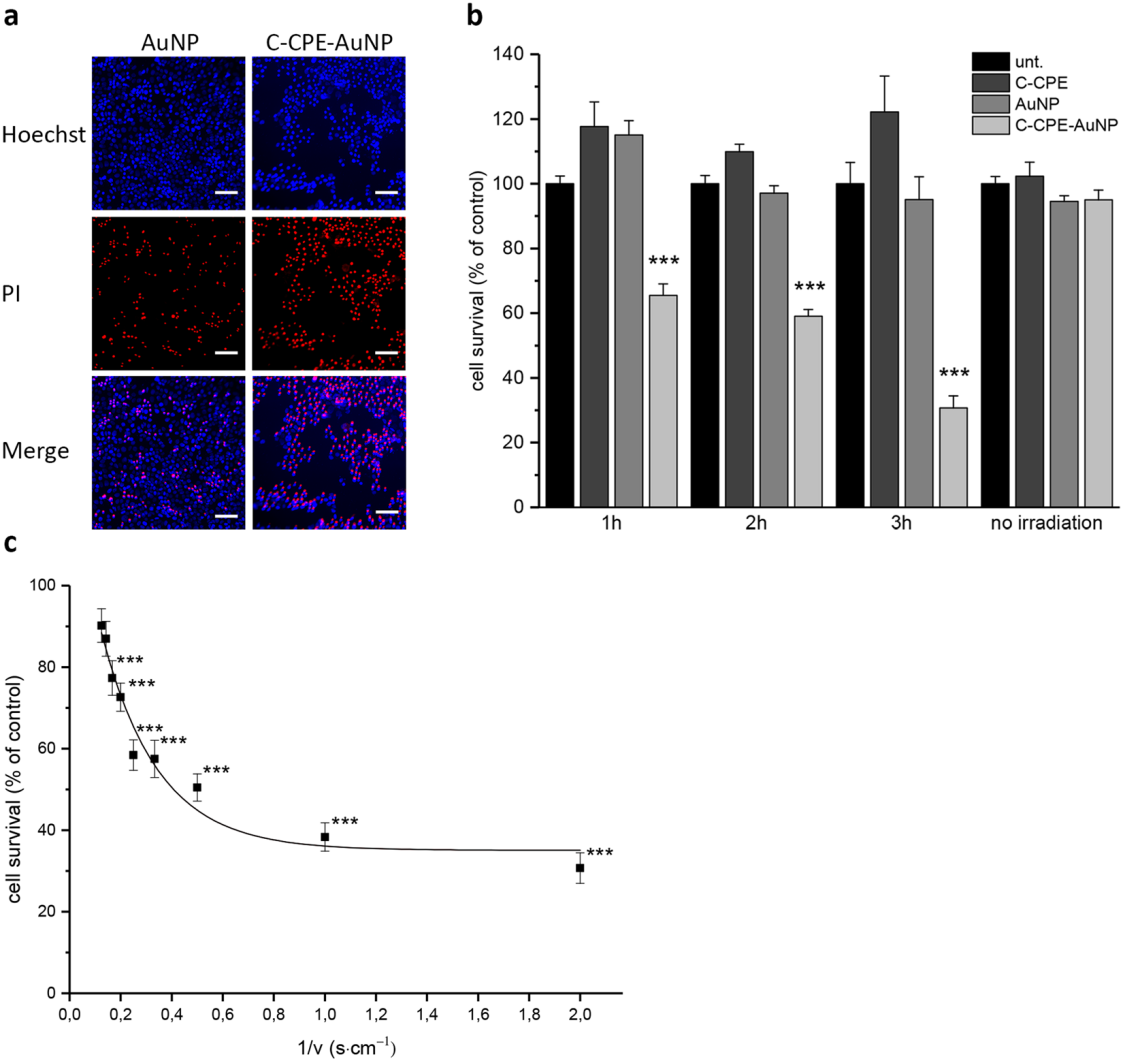
Killing tumor cells using gold nanoparticles functionalized with C-CPE (C-CPE-AuNP). (**a**) Propidium iodide (PI) uptake as indicator of cell death of Caco-2 cells after GNOME-LP application (60 mJ/cm^2^ at 0.5 cm/s). The micrographs show an increased PI uptake in cells treated with GNOME-LP in combination with C-CPE-AuNP complex compared to cells treated with GNOME-LP in combination with non-functionalized AuNP. All nuclei are stained with Hoechst (blue). PI uptake (killing) is shown in red. Scale bar, 100 µm. (**b**) Increased efficiency of C-CPE-AuNP complex mediated cell killing by prolonged adhesion time of the complex onto the cells. (**c**) The efficiency of cell killing was also enhanced by reduction of the scanning speed as shown in the plot of the relative cell survival against the reverse of respective scanning speed. All graphs represent the mean ± SEM of cell survival relative to untreated cells as control reference. Results analyzed with Student’s t test. *Significant difference to the control reference: ***P < 0.001.

In order to test the applicability of GNOME-LP mediated killing by the C-CPE-AuNP complex, Strep-Tactin conjugated 25 nm AuNPs were mixed with the C-CPE construct at a final concentration of 2.5 · 10^10^ AuNPs/ml and 5 µg/ml C-CPE. The C-CPE concentration was chosen after we observed that the 5 µg/mL was enough for binding to claudin expressing cells and destabilization of the barrier function of a Caco-2 monolayer (Fig. 3a). The C-CPE-AuNP complex was applied to the Caco-2 cells and the setting parameters of GNOME-LP were tested by observing whether the treatment induced propidium iodid uptake (Fig. 4a). Detection of promidium iodid uptake was considered as indicator of cell death. The counting of cells stained with Hoechst and propidium iodid allowed to estimate the killing efficiency. Cells stained with Hoechst but not with propidium iodid were considered as not killed. First, the GNOME-LP was applied at the maximal laser fluence (60 mJ/cm^2^) at a scanning speed of 0.5 cm/s on cells that were incubated with the C-CPE-AuNP complex for different time points to allow complex adhesion to the cells surface.

As shown in Fig. 4b, the survival of Caco-2 cells decreased with the duration of the adhesion time, achieving a minimal survival of less than 30% for an adhesion time of 3 h. Further prolongation of the incubation time did not increase the killing effect (data not shown). Therefore, an adhesion time of 3 h was chosen for further experiments. In control groups, treated either with AuNP or C-CPE alone, GNOME-LP application did not impair cell survival (Fig. 4b). Further, we observed that the killing efficiency was inversely dependent on the scanning speed. Application of a constant laser fluence of 60 mJ/cm^2^ at scanning speed from 8 cm/s to 0.5 cm/s increased the killing efficiency. To analyze this dependency, the relative cell survival was plotted against the reverse of the respective scanning speed. Using the non-linear curve fit assistant of OriginPro 2017, the data points were fitted to the exponential equation (Fig. 4c).

$$y=a+(b-a){e}^{(-\frac{\alpha -{\alpha }_{th}}{k})}$$

**Figure 5:**
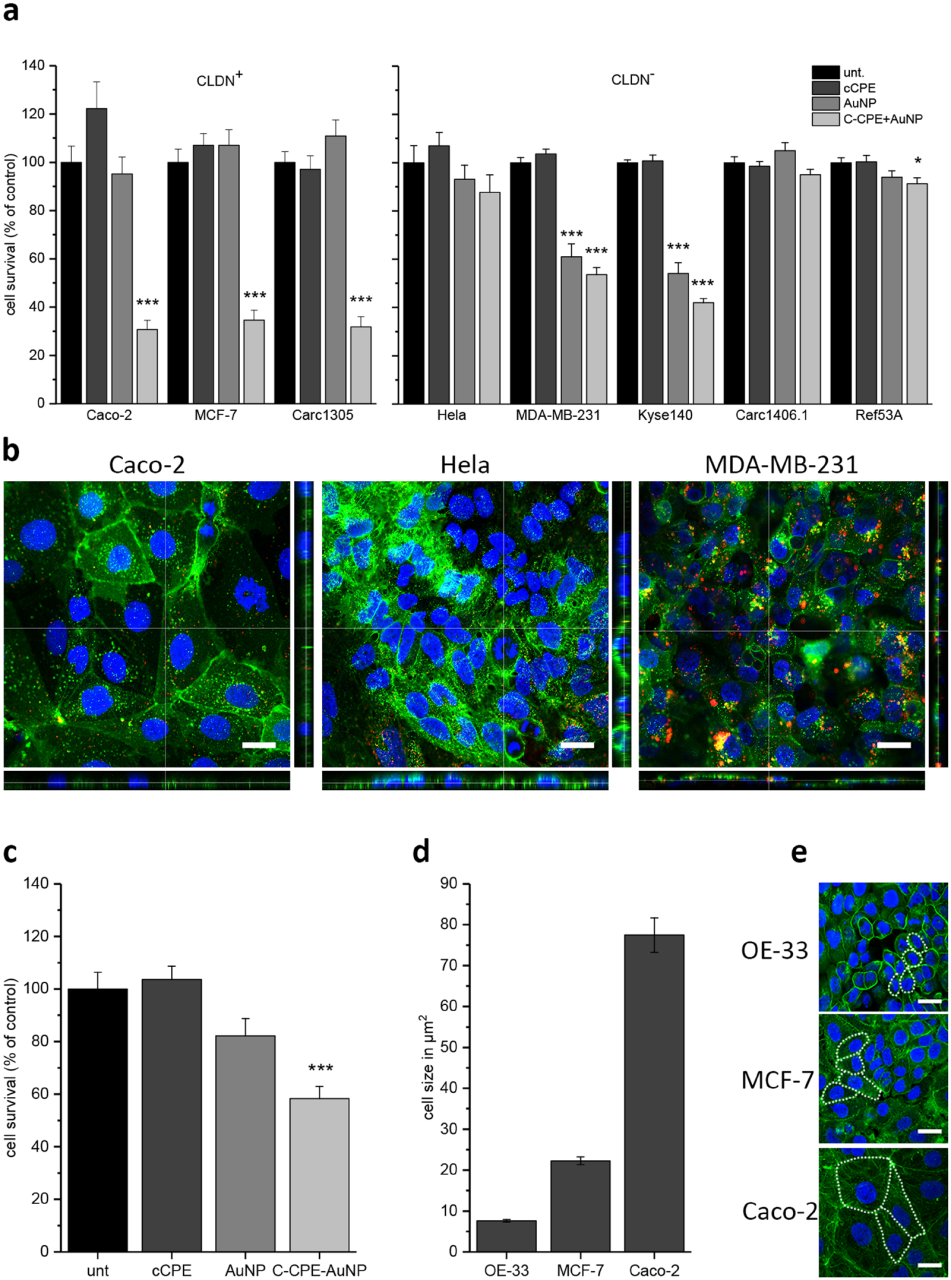
Killing effect of different tumor cells with C-CPE functionalized AuNP. (**a**) GNOME-LP (60 mJ/cm² at 0.5 cm/s) applied in combination with C-CPE-AuNP reduced cell survival of the claudin expressing Caco-2, MCF-7 and Carc1503 cells. The claudin non-expressing Hela, Carc1406.1 and Ref53A were not affected. Note the reduction of cell survival of the claudin non-expressing MDA-MB-231 and Kyse140 cells. For these cells, the reduction of cell survival was also achieved by non-functionalized AuNP. Graphs represent the mean ± SEM of cell survival relative to untreated cells as control reference. The results were analyzed with Student’s t test. *Significant difference to the control reference: *P < 0.05 and ***P < 0.001. (**b**) Confocal fluorescence images showing endocytosis of 40 nm nanobeads (red) by Caco-2, MDA-MB-231, and Hela cells. In MDA-MB-231 cells more beads (red) and even beads contained in endocytosis vesicles (yellow) were detected compared to Caco-2 or Hela cells. Cell membranes were stained with CellBrite (green) and Nuclei with Hoechst (blue). Scale bar, 25 µm. (**c**) Application of GNOME-LP in combination with the C-CPE-AuNP complex increased the reduced OE-33 cell survival compared to non-functionalized AuNPs. Graphs represent the mean ± SEM of cell survival relative to untreated cells as control reference. The results were analyzed with Student’s t test. *Significant difference to the control reference: ***P < 0.001. (**d**) Different cell size of OE-33, MCF-7 and Caco-2. Graphs represent the mean ± SEM of area size. (**e**) Exemplary micrographs showing the area (dotted lines) of OE-33, MCF-7 and Caco-2 cells. Staining of F-Actin with Phalloidin iFluor 488 (green) and Nuclei stained with Hoechst (blue). Scale bar, 100 µm.

From the equation, a scanning speed for half-maximal efficiency (*k*) of 5 cm/s was estimated. In the above equation “*y*” gives the relative amount of cells, which survived the killing for each scanning speed. “*b*” is the relative amount of cell survival (100%) when a GNOME-LP was not applied. “*a*” is the relative amount of cells which survived the treatment at minimal scanning speed (0.5 cm/s). α gives the reverse of the scanning speed, and α_th_ is the reverse of the maximal scanning speed (0.15 s/cm^−1^) at which a significant cell killing was observed. For further analyses, we applied a laser fluence of 60 mJ/cm^2^ and a scanning speed of 0.5 cm/s to the other cells types after an adhesion time of 3 h to test the efficiency of the killing method. Laser exposure of claudin expressing cells such as the human MCF-7 cells or the canine TiHoDMglCarc1305 cells decreased the cell survival up to 30% (Fig. 5a). For the OE-33 cells, GNOME-LP in combination with C-CPE functionalized AuNPs reduced the cell survival by about 40%, GNOME-LP in combination with non-functionalized AuNPs did not affect the cell survival. The reduced cell killing efficiency may be related to the size of OE-33 cells. Schomaker *et al*.^42^ showed that, small cells were less sensitive to GNOME-LP as compared to large cells. As shown in Fig. 5d OE-33 cells were small in comparison to MCF7 or Caco-2 cells. In general, no significantly decreased cell survival of the claudin non-expressing human Hela or the canine TiHoDMgl1406.1 and TiHoDMgIRef53A cells was observed after the treatment of the C-CPE-AuNP complex combined with the GNOME-LP. In those cells, the application of GNOME-LP in presence of the C-CPE-AuNP complex killed about 10% of the cells. The results are comparable to that found when GNOME-LP was applied in combination with AuNP or C-CPE alone (Fig. 5a). For the claudin non-expressing human MDA-MB-231 and Kyse140 cells the GNOME-LP treatment in combination with AuNPs without C-CPE killed 39% and 46% cells respectively. This killing efficiency was not significantly increased when GNOME-LP was combined with the C-CPE-AuNP complex. The killing of AuNP treated MDA-MB-231 cells and Kyse140 cells may be related to endocytotic activity of these cells allowing them to internalize the AuNPs. The high endocytotic activity of the MDA-MB-231 cells could be experimentally shown with fluorescent nanobeads (40 nm). Compared to Caco-2 or Hela cells, MDA-MB-231 internalized more nanobeads (Fig. 5b). Taken together, the result show that the functionalization of AuNPs with C-CPE increases the killing efficiency and specificity of tumor cells expressing the relevant claudin proteins.

### Application of the C-CPE-AuNP complex for an optic induced killing of 3D tumor spheroid models

**Figure 6:**
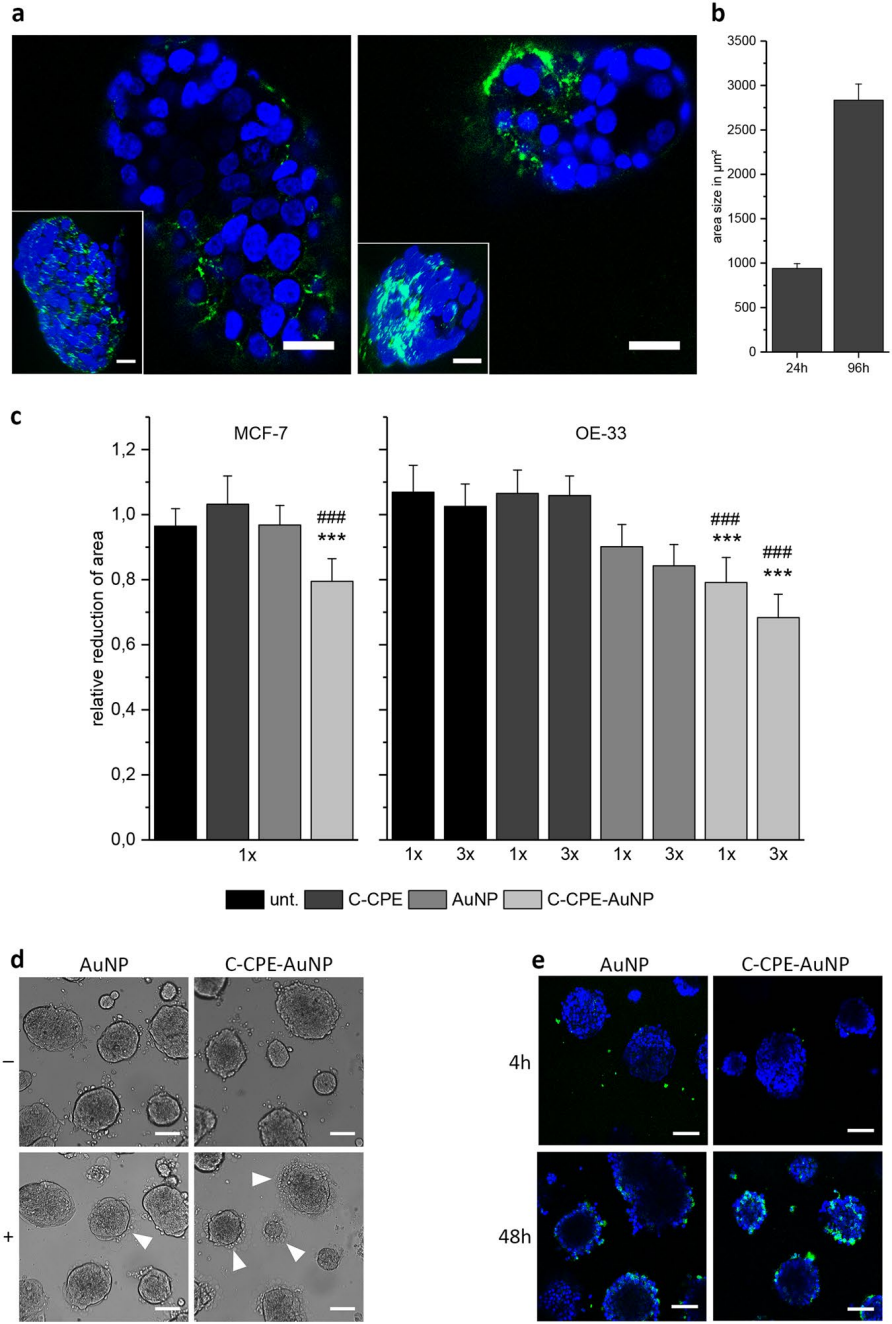
Killing of cells grown in spheroids with C-CPE Functionalized AuNPs. (**a**) Spheroids formed by OE-33 sand MCF-7 cells cultivated in Matrigel. Confocal images of spheroids showing C-CPE-Strep-Tactin Chromeo 488 (green) binding at cell-cell contacts. The inserts are reconstituted images of the spheroids after Z-stack recordings. Nuclei are stained with Hoechst (blue). Scale bar, 25 µm. (**b**) OE-33 spheroids proliferate in Matrigel indicated by increased spheroid area size over cultivation time. The spheroids were imaged in the same plane 24 h and 96 h after seeding. Graphs represent the mean ± SEM of area size. (**c**) Quantitative evaluation of the spheroid area reduction by combination of GNOME-LP with non-functionalized AuNPs or the C-CPE-AuNP complex. The C-CPE-AuNP complex increased the efficiency to reduce the spheroid area of MCF-7 and OE-33. Multiple GNOME-LP application (3x) increased the reduction of the OE-33 spheroid area. Graphs represent the mean ± SEM of the spheroid area reduction relative to the spheroid area before laser irradiation. The results were analyzed with Student’s *t* test. *Significant difference to the untreated spheroids: ***P < 0.001 and ^#*##*^*P* < 0.001 significant difference to the AuNP treated spheroids. (**d**) Exemplary micrographs showing OE-33 spheroids before (−) and after (+) GNOME-LP application with the C-CPE-AuNP complex or non-functionalized AuNPs. The presence of the C-CPE-AuNP complex reduced spheroid area and disrupted spheroid boundaries indicated by arrow heads. Scale bar, 100 µm. (**e**) Annexin V staining (green) of non-functionalized AuNPs and C-CPE-AuNP treated OE-33 spheroids after 4 h and 48 h threefold GNOME-LP applications. The C-CPE-AuNP complex in combination with GNOME-LP induced cell apoptosis after 48 h and smaller spheroids compared to non-functionalized AuNPs treated spheroids. Nuclei are stained with Hoechst (blue). Scale bar, 100 µm.

Tumors are 3D cell systems with an extracellular matrix maintaining the cells together. For the cell killing within a tumor, the C-CPE-AuNP complex must be able to diffuse through the matrix and bind to the cells. We therefore evaluated whether cells in extracellular matrix are still accessible for the C-CPE-AuNP complex. Cultivated in Matrigel, OE33 and MCF-7 cells formed spheroids whose volume increased with the cultivation duration (Fig. 6a,b). Within the spheroids, the cells expressed claudins as revealed by staining with Strep-Tactin Chromeo 488 conjugated to C-CPE. In the staining experiments, claudin was mostly found as expected in cell-cell junctions (Fig. 6a). The data showed that the cells within 3D spheroids correctly express the claudin proteins. The results further suggest that the C-CPE was able to enter the Matrigel and to specifically bind to claudin proteins in the cell-cell junctions. Therefore, we tested the usability of the C-CPE-AuNP complex combined with GNOME-LP for spheroid killing. The C-CPE-AuNP complex, C-CPE, or AuNP alone were given to the spheroids. After a diffusion and adhesion time of 3 h, microscopic images of the spheroids were taken followed by GNOME-LP (60 mJ/cm², 0.5 cm/s) treatment (Fig. 6c). Images were again taken 2–4 h after the GNOME-LP treatment in exactly the same plane of spheroids as before GNOME-LP application to allow a comparison of the spheroid areas before and after GNOME-LP treatment. The results indicated that GNOME-LP in combination with the C-CPE-AuNP complex reduced the spheroid area (Fig. 6d). The spheroids treated with AuNP alone in combination with GNOME-LP showed less pronounced reduced area compared to spheroids treated with GNOME-LP combined with the C-CPE-AuNP complex. The images of spheroids after GNOME-LP with the C-CPE-AuNP complex application showed additionally destroyed spheroid boundaries, probable due to cell death (Fig. 6c). The killing efficiency was increased by multiple laser exposure suggesting that a multiple laser treatment of tumor spheroids could result in a total ablation (Fig. 6d). Furthermore, by staining the apoptosis marker annexin V 48 h after laser treatment, we found an increased apoptosis in the spheroids treated with GNOME-LP combined with the C-CPE-AuNP complex as compared to GNOME-LP combined with the non-functionalized AuNPs (Fig. 6e), suggesting a long time effect of the GNOME-LP combined with the C-CPE-AuNP complex. Collectively, the results clearly indicate that the C-CPE-AuNP complex increased the killing efficiency of the GNOME-LP technique in an *in vitro* 3D culture system.

## Discussion

The present report shows that C-CPE coupled to Strep-Tactin conjugated gold nanoparticles (C-CPE-AuNP) can be combined with the gold nanoparticle-mediated laser perforation (GNOME-LP) technique^32^ for a specific targeting of claudin-3, -4 and 7 expressing tumor cells (Figs 1, 4 and 5).

Many tumor types such as colon and breast tumor, are characterized by a reinforced expression of the CPE receptors claudin-3 -4 or -7^23,24,43–45^ (Fig. 2). It was therefore proposed to use CPE for tumor diagnosis and therapy^46–50^. Previous experiments demonstrated that CPE by binding to a subset of claudins, e.g. claudin-3 and -4, killed cancer cells^15–17^. Since a systemic application of CPE to kill tumor xenograft caused deleterious side effects^27^ another way to use the enterotoxin for tumor killing should be developed. We suggest to use the non-cytotoxic C-terminal domain (D194-F319), which binds to claudins^51,52^, for functionalization of a cell destroying component e.g. gold nanoparticles (Fig. 1). The AuNPs can then be specifically targeted on claudin expressing cells e.g. tumor cells. Optical method to activate the AuNPs can be applied for the tumor ablation. In this context, C-CPE could be a tool in cancer therapy with less side-effects compared to the original CPE. The technique could be a complement or an alternative to antibodies functionalized AuNPs^14,53–55^.

Our observations show that the Strep-Tag fused to the N-terminal domain of our C-CPE construct does not alter the protein function and thus we used the C-CPE construct to functionalize gold nanoparticles. The decreased TEER (Fig. 3a) of expressing cells Caco-2 cell monolayer as well as of the MCF-7, OE-33 and TiHoDMglCarc1503 cell monolayer (data not shown) without affecting cell viability (Fig. 3b) suggests that the C-CPE specifically targeted claudin proteins causing a modulated tight junction integrity (Table 1). Therefore, C-CPE is not per se cytotoxic and might cause less harmful side effects. Concerning the binding affinity of C-CPE to specific claudins, C-CPE binds to claudin-3 and claudin-4 with a K_d_ value around 1 × 10^8^ M^−1^ estimated using an optimized assay with claudin-3 and claudin-4 transfected cells^22^. For the 15 kDa C-CPE, 10^8^ M^−1^ correspond to a concentration of about 1.2 µg/ml. In our experiments, we used a C-CPE concentration of 5 µg/ml. This concentration is in the concentration range, which was used by other authors (4–10 µg/ml) for cells naturally expressing the C-CPE receptors *in vitro* and *in vivo*^33,34^. The reduced sensitivity to C-CPE is possible due to a different expression of the proteins in native cells as compared to transfected cells. However, the data showed that our recombinant C-CPE bound to its receptors in the cell membrane. The Strep-Tag was used to functionalize chromophore labeled Strep-Tactin for imaging claudin expressing cells or Strep-Tactin conjugated gold nanoparticles for tumor cell killing. Imaging of C-CPE binding to tumor cells (Fig. 3c,d) confirmed that the protein is capable to specifically target its receptors claudin-3, -4 and -7 expressed in the relevant tumor cells and demonstrated that the functionalization did not alter the binding capacity to the claudins.

**Table 1:** Result summary of claudin (CLDN) expression, C-CPE binding and cell killing of human and canine cells.

Cell line	Species	Tissue	CLDN expression	C-CPE binding	C-CPE effect on TEER	Cell killing
Caco-2	human	colon adenocarcinoma	CLDN-3, -4, -7	+	↓	+
MCF-7	human	mammary adenocarcinoma	CLDN-3, -4, -7	+	↓	+
MDA-MB-231	human	mammary adenocarcinoma	−	−	−	+
OE-33	human	Esophegal adenocarcinoma	CLDN-3, -4, -7	+	↓	+
Kyse140	human	Esophegal squamous carcinoma	−	−	−	+
Hela	human	cervix carcinoma	−	−	−	−
TiHoDMglCarc1305	canine	complex adenoma	CLDN-3, and -4	+	↓	+
TiHoDMglCarc1406.1	canine	carcinoma complex type	−	−	−	−
TiHoDMgl Ref53A	canine	non-neoplastic mammary tissue	−	−	−	−

The GNOME-LP technology was previously found as a high-throughput, cell friendly and rapid method for a transient cell permeabilization allowing an uptake of molecules, like dyes or siRNA into cells^32,35,36,56^. The gentle cell permeabilization was achieved by heat generated through the interaction of laser pulses and gold nanoparticles randomly adherent on the cells. In these experiments, gold nanoparticles and energy density of the applied laser beam were adjusted to achieve a transient permeabilization^32,42,56^. In consideration of the survival, patch-clamp experiments suggested that the repair of the cells after GNOME-LP treatment had two components. (i) The closure of a GNOME-LP induced pore that has to take place within about 10 s after GNOME-LP application. (ii) A slow (about 20 min) rearrangement of the lipid bilayer in the former pore to build a totally intact membrane. During the repair process, an uptake of small molecules from the external milieu is possible^42,57^. The survival depends on a success of these repair process. To analyze killing efficiency of GNOME-LP application using PI uptake, it is important to give PI in the cell milieu after the repair process. Evidently, the survival of cells could be strongly reduced by increasing the energy density of the applied laser beam^58–60^. In our cell treatment, the GNOME-LP at a fluence of 60 mJ/cm^2^ and a scanning speed of 0.5 cm/s in combination with large AuNP (200 nm) without functionalization clearly reduced cell survival to about 50% (data not shown). However, the efficiency and particularly the specificity were lower compared to tumor cell killing using C-CPE functionalized gold nanoparticles (Fig. 5a). The functionalizing of AuNPs with C-CPE (C-CPE-AuNP) reduced the cell survival to less than 30% of claudin expressing Caco-2, MCF-7 and TiHoDMglCarc1305, while the exposure of claudin non-expressing cells such as Hela, TiHoDMglCarc1406.1 and TiHoDMglRef53A did not affected the cell survival (Fig. 5a). The increased cell killing efficiency could, at least partly, be related to the fact that the 25 nm (Ø) AuNPs have an optimal absorbance at 527 nm^61,62^, which is very close to the used laser wavelength (532 nm). Moreover, nanoparticles with reduced diameter may be appropriated for cell killing *in vivo*, since thy may diffuse better into tumors. The increase of specificity is solely due to the close binding of the AuNPs on claudin expressing cells via the hook constituted by C-CPE. Of note, firstly, the claudin expressing OE-33 cells seemed to be less tackled by the combination of GNOME-LP and the C-CPE-AuNP complex (Fig. 5a). Schomaker *et al*.^42^ reported that plasmon mediated cell permeabilization efficiency was related to the cell size. Thus, the reduced cell killing efficiency OE-33 cells could be due to their small size in comparison to MCF-7 or Caco-2 cells (Fig. 5d,e). Secondly, the claudin non-expressing cells such as MDA-MB-231 or Kyse140 were significantly killed by GNOME-LP (60 mJ/cm^2^, 0.5 cm/s) combined with C-CPE-AuNP or AuNPs (Fig. 5a) possibly due to elevated endocytotic activity of these cells (Fig. 5b). However, the observation that C-CPE functionalized AuNPs increased killing of claudin expressing cells up to more than 75% revealed the powerful efficiency of the AuNP functionalization by C-CPE. Killing tumor cells by optical methods was also shown when gold nanoparticles were functionalized using antibodies against EGF- or TF- receptorsel^13,14^. The data in this report show that C-CPE could represent an easy and competitive complement or alternative functionalization method to combat tumors. With respect to tumors, the data produced using the esophageal adenocarcinoma OE-33 and the mammary adenocarcinoma MCF-7 cells cultivated in 3D culture systems showed that C-CPE was still able to recognize the claudins at the cell membrane within the 3D structures (Fig. 6a) and to link gold nanoparticles in a tissue-like system. OE-33 and MCF-7 cells are particularly interesting cells models, which were largely used as xenografts and constitute good tumor models^63,64^. Mentionable is the reduced killing efficiency of the C-CPE-AuNP complex and that non-functionalized AuNP achieved a non-negligible killing level (Fig. 6d). This is probable due to non-specific retention of the gold nanoparticles in the matrix, which might take place in tissues. However, our results showed that when gold nanoparticle were functionalized with C-CPE, a significant more elevated killing of the cells within the matrix compared to non-functionalized gold nanoparticles. However, we observed a reduced killing efficiency in the 3D culture system compared to cells growing in monolayer using a laser fluence of 60 mJ/cm^2^ and a scanning speed of 0.5 cm/s. It is possible that the Matrigel caused a loss of energy. We can also not exclude that the cells in 3D may express less claudins as compared to the cells cultivated in 2D, although the imaging using chromeo 488 dye labeled C-CPE did not show a strong reduction of claudin expression (Figs 3c and 6a). The possible energy loss because of the matrix can be compensated by a multiple application of the GNOME-LP, where a threefold application of GNOME-LP increased the killing efficiency (Fig. 6c). Interestingly, we could show that the combination of GNOME-LP and C-CPE functionalized AuNPs had a long time effect characterized by a continuous apoptosis (Fig. 6e) suggesting that even cells that were not directly destroyed, were irreparable injured by the treatment. Moreover, the use of near-infrared (NIR) laser pulses (650–900 nm) which are less absorbed by tissue^64^ could be an option to improve the cell killing in tumor tissues and tissue-like structures. For instance, the co-application of NIR laser pulses with the C-CPE-AuNP complex could be used for cutaneous neoplasia. Furthermore, endoscopic guided laser ablation in combination of pre-treated C-CPE-AuNP tumors could be a strategy to specific combat chemo-resistant tumor types overexpressing the C-CPE receptor claudin-3 and -4 without adverse side effects.

## Conclusion

Our observations show that C-CPE can be used to functionalize gold nanoparticles in order to specifically and efficiently kill a broad spectrum of claudin expressing tumor cells using an optical device such as the GNOME-LP (Fig. 1, Table 1). The method can be used even when cells proliferate in Matrigel showing that the method could be an opportunity to target tumors in tissues during a therapeutic intervention. The report also demonstrates that C-CPE can be linked to a chromophore labeled Strep-Tactin allowing the visualization of tumor cells expressing claudins. For some cancers, metastatic cells express claudin proteins. Therefore, the C-CPE coupled to a chromophore or AuNPs could be an interesting tool in cancer diagnosis and even therapy used for detection or killing of metastatic cells in body fluids.

## Materials and Methods

### Preparation of the *C. perfringens* enterotoxin C-terminal fragment (C-CPE)

The coding region of C-CPE_194–319_ protein (NCBI Acc. Nr. M98037.1) was amplified from the genomic *C. perfringens* DNA (NCTC8239, DSMZ, Braunschweig, Germany) using specific primers (Table 2). Subsequently, the amplified *c-cpe* gene was purified for TA-cloning in pGEM-T easy vector (Promega). After plasmid isolation, the *c-cpe* gene fragment was amplified using primers with *NdeI* and *XhoI* restriction sites (Table 2). For protein expression in *E. coli* Rosetta pLysSRARE2 (Novagen, Merck, Darmstadt, Germany) the *c-cpe* gene was inserted in the expression vector pet22b using *XhoI* and *NdeI* restriction sites, which allowed fusion of the c-cpe gene with the Strep II-Tag. The correct insertion of the gene in the vector (pet22b-strep-c-cpe) was verified by sequencing. After transformation in *E. coli* Rosetta, the expression of C-CPE protein was induced by adding 1 mM IPTG. Thereafter, the cells were collected, lysed and the Strep II-Tag fusion C-CPE was isolated and purified using the Strep-Tactin Superflow column (IBA, Göttingen, Germany). The elution buffer consisting of (in mM) 150 Nacl, 1 EDTA, 2.5 Desthiobiotin, 100 Tris/HCl (pH: 8) was used to separate Strep-C-CPE from the column. The product was quantified by photometry and verified by western blot using the Strep-Tactin HRP antibody (1:4000).

**Table 2:** Primer sequences used for C-CPE production. Restriction site *NdeI* of forward primer (Frw) and *XhoI* of reverse primer (Rev) are underlined.

Target gene	Primer sequence 5′-3′	
CPE	Frw Rev	GAAAGATGTGTTTTAACAGTTCCATCTACA TTAAAATTTTTGAAATAATATTGAATAAGGGTAATTTCCACTTA
C-CPE	Frw Rev	GCGCATATGGATATAGAAAAAGAAATCCTTGATTTAGC GCCTCAAGAAATTTTTGAAATAATATTGAATAAGGG

### Cell Culture

Human tumor cell lines Caco-2, MCF-7, Hela (DSMZ, Braunschweig, Germany), OE-33, Kyse140, MDA-MB-231 as well as canine tumor cell lines^39,40^ TiHoDMglCarc1305 (former called T120A,), TiHoDMglCarc1406.1 (former called DT14/06T) and TiHoDMglRef53A (former called MTH53A) were grown in tissue coated petri dishes at 37 °C in a humidified atmosphere containing 5% CO_2_. The human cell lines and canine cell lines were cultured in, respectively, DMEM Ham’s F12 (Biochrom) and M199 medium (Sigma) supplemented with 10% fetal calf serum, 100 units/mL penicillin and 100 µg/mL streptomycin.

### Spheroid formation

Spheroids formed by OE-33 or MCF-7 cells grown in growth factor reduced Matrigel (Corning). Briefly, 60 µl Matrigel were given in a well of a µ-Plate 96 well (ibidi, Martinsried, Germany). The plate was placed in cell culture incubator for 30 min to allow gel formation. Next, the Matrigel was overlaid with cell culture medium (250 µl). Then cells were added in the wells (5 · 10^4^ cells per well) and cultured for 3–4 days to allow spheroid formation.

### Cell viability

The effect of C-CPE on cell viability was assessed using MTT assay. Briefly, cells were grown as monolayer in a 96 well plate. Subsequently, cells were incubated for 24 h with C-CPE. MTT reagent (0.5 µg/ml in PBS) was added to each well and incubated for 4 h at 37 °C. The formed formazan crystals were lysed in 100 µl isopropyl containing 40 mM hydrochloric acid. The absorbance was measured at 560 nm in a microplate reader (Mithras LB940, Berthold Technologies, Germany).

### Formation of C-CPE-Chromeo 488 and C-CPE-AuNP complex

For visualization the claudins in cells the C-CPE-Chromeo 488 complex as well as for tumor cell killing the C-CPE-AuNP complex were freshly prepared before usage. The C-CPE-Chromeo 488 complex was formed by mixing 2.5 µl Strep-Tactin® Chromeo™ 488 as provided by the manufacturer (iba, Göttingen; Germany) with C-CPE in elution buffer. The mix was incubated overnight at 4 °C to allow a binding between C-CPE and Strep-Tactin® Chromeo™ 488. Thereafter, cell culture medium was added achieving a working concentration of 5 µg/ml C-CPE.

For the C-CPE-AuNP complex, Strep-Tactin®-AuNP were produced by conjugation of Strep-Tactin (iba) with 25 nm AuNP (Aurion, Wageningen, Netherlands). To produce the C-CPE-AuNP complex, C-CPE in elution buffer and Strep-Tactin®-AuNP, as provided by the manufacturer, were mixed and incubated overnight at 4 C. Cell culture medium was added to achieve a working volume of 100 µl for each well of a µ-Plate 96. The volume of the C-CPE andStrep-Tactin®-AuNP solution in mixture were ajusted to a working concentrations of 5 µg/ml C-CPE and 2.5 · 10^10^ AuNPs.

### Monitoring of transepithelial electrical resistance (TEER)

TEER measurement was performed by impedance spectroscopy using the cellZscope (nanoAnalytics, Muenster, Germany). For measurement, human or canine cells were seeded in inserts consisting of a transparent PET membrane with pore size of 0.4 µm (Cat. No: 353504; BD Falcon, Corning) in DMEM or M199 cell culture medium. Inserts with the cells were transferred into the cellZscope and placed in the cell culture incubator. TEER data were automatically registered every 3 h with the cellZscope software. Once cells reached a constant TEER, cells were treated with C-CPE diluted in cell culture medium and TEER was monitored every hour.

### Real-time PCR

The level of mRNA expression of human claudin-3, -4 and -7 (CLDN-3, -4 and -7) was examined by quantitative real-time PCR. Briefly, the total RNA was isolated using the peqGold Total RNA kit (PEQLAB Biotechnologie GmbH, Erlangen, Germany) according to the manufacturer’s protocol. The following cDNA synthesis was performed with the maximal first strand cDNA synthesis kit (ThermoScientific). Primer pairs for CLDN-1, CLDN-3, CLDN-4 and CLDN-7 (Table 3) were designed according to the mRNA sequences given in the National Center for Biotechnology Information (NCBI). The amplificates were sequenced to confirm all genes. For real-time PCR 1 x Kapa SYBR FAST Universal mastermix (Sigma Aldrich) was used and PCR reaction (40 cycles followed by a melting stage) was performed in the real-time PCR cycler peqSTAR 96q (PEQLAB Biotechnologie GmbH). Gene expression was normalized to endogenous housekeeping genes 3-phosphate dehydrogenase (GAPDH) and beta‐actin (ACTB), ratio of gene expression based on 2^−ΔΔCt^.

**Table 3:** List of primer sequences used for real-time PCR.

Target gene	NCBI AccNo.	Primer sequence 5′-3′		Amplicon size (bp)
ACTB	NM_001101.4	Frw Rev	CCTTGCACATGCCGGAG GCACAGAGCCTCGCCTT	112
GAPDH	NM_001256799.2	Frw Rev	TTGAGGTCAATGAAGGGGTC GAAGGTGAAGGTCGGAGTCA	117
CLDN-3	NM_001306.3	Frw Rev	CCAACCTGCATGGACTGTGA TCGACGGGGTGGTCAAGTAT	80
CLDN-4	NM_001305.4	Frw Rev	GGCCTATGGATGAACTGCGT AGCCACGATGATGCTGATGA	129
CLDN-7	NM_001185022	Frw Rev	GGACAGTGGGTCGCCG CACAAATCGACCCCTCCAGT	138

### Visualization of claudins in cells

Expressed claudin-3, -4 and -7 in cells were visualized with immunofluorescence or the C-CPE-Chromeo 488 complex. For immunofluorescence, the cells were grown to monolayer on rat collagen type I coated cover slips. After washing with PBS, the cells were fixed with 4% formaldehyde and permeabilized with 0.3% TritonX-100 in PBS. After washing with PBS unspecific binding sites were blocked with 1% BSA in PBS for 30 min at 37 °C. Claudins were stained with primary antibodies (Table 4) diluted in PBS with 1% BSA overnight at 4 °C. Subsequently, the cells were washed with PBS and incubated with fluorescein conjugated anti-rabbit (Merck Millipore) or FITC conjugated anti-mouse secondary antibody (Merck Millipore, AQ303F) diluted 1:100 in PBS with 1% BSA (SigmaAldrich) for 1 h at 37 °C. For nuclear staining, DAPI (2 µM) was added during the staining with secondary antibody. For C-CPE-Chromeo 488 complex related claudin visualization, cells similarly cultured as for immunofluorescence, were stained with C-CPE-Chromeo 488 complex as described above. The cells were stained for 3 h in a cell culture incubator. Nuclei were stained with 1 µM Hoechst added to the cells for the last incubation hour. The cells were fixed with 4% formaldehyde, washed with PBS, and stored in PBS for further confocal microscopic analysis.

**Table 4:** Antibodies used for immunofluorescence experiments.

Protein	Antibody	Working concentration
CLDN-1	rabbit anti-human CLDN-1 (Thermo Fischer Scientific, 59–9000)	5 µg/ml
CLDN-3	Rabbit anti-mouse CLDN-3 (Thermo Fischer Scientific, 34–1700)	3 µg/ml
CLDN-4	Mouse anti-human CLDN-4 (Thermo Fischer Scientific, 34–1700)	3 µg/ml
CLDN-7	Rabbit ant-human CLDN-7 (Thermo Fischer Scientific, 32–9400)	2 µg/ml

### Gold nanoparticle-mediated (GNOME) laser perforation for tumor cell killing

For tumor cell killing confluent cells or spheroids were treated with the C-CPE-AuNP complex for 3 h in cell culture incubator to allow complex adhesion to the cells. Control groups were incubated with either non-functionalized AuNPs or C-CPE alone. The µ-96 plate with the cells was transferred to the laser perforator (Laser Zentrum Hannover, Hannover, Germany) as described in Heinmann *et al*.^32^, and exposed to a 20 kHz pulsed laser (532 nm) with 60 mW (60 mJ/cm²) at 0.5 cm/s. After optical treatment, the cells were transferred back into the incubator for 30–60 min to allow not killed cells to reseal the membrane as it was shown that only permeabilized but not killed cells were able to reseal the membrane within 20 min^42,57^. After optical treatment, the cells were transferred back into the incubator for 30–60 min. Propidium iodide (10 µM) and Hoechst 33258 (Sigma Aldrich) were added to the cells for 60 min. Under fluorescence microscope, dead cells were indicated by propidium iodide and Hoechst uptake, while health cells were only Hoechst positive. Apoptotic cells were indicated by annexin V ATTO 488 conjugate (1:40 in cell culture medium) staining.

### Cell Imaging

Immunofluorescence, C-CPE-Chromeo 488 binding or nanobead endocytosis was imaged with Nikon Eclipse TE2000-E confocal laser scanning microscope (461 nm for DAPI/Hoechst, 488 for C-CPE/CLDN/CellBrite/annexin V and 535 nm for nanobeads), with a 60x water immersion objective and EZ-C1 3.80 software program (Nikon, Düsseldorf, Germany).

Propidium iodide (PI) uptake after cell killing was performed with a Ti-E inverted fluorescence microscope (Nikon, Duesseldorf, Germany). Images were taken with 10x objective and Nikon Software Nis-Elements 4.4 (346 nm for Hoechst 33258 and 535 nm for PI). All images were processed with ImageJ/Fiji for further quantitative evaluation.

### Statistical analysis

The result are given as mean of at least three independent experiments for each treatment. The error bars represent S.E.M. Statistical comparison between groups was performed using the Student’s paired two-sided t test.
