# Publisher Correction: Functionalization of gold-nanoparticles by the *Clostridium perfringens* enterotoxin C-terminus for tumor cell ablation using the gold nanoparticle-mediated laser perforation technique

**DOI:** 10.1038/s41598-018-37180-8

**Published:** 2019-03-06

**Authors:** Annegret Becker, Miriam Leskau, Barbara L. Schlingmann-Molina, Susanne C. Hohmeier, Suhayla Alnajjar, Hugo Murua Escobar, Anaclet Ngezahayo

**Affiliations:** 1 Institute of Cell Biology and Biophysics, Department of Biophysics and Cell Physiology, Leibniz University Hannover, Hannover, Germany; 2 Meissa Vaccines, South San Francisco, California, USA; 3 Small Animal Clinic, University of Veterinary Medicine Hannover, Hannover, Germany; 4 Division of Medicine, Haematology, Oncology and Palliative Medicine, University of Rostock, Rostock, Germany; 5 Center of System Neuroscience, University of Veterinary Medicine Hannover, Hannover, Germany

Correction to: *Scientific Reports* 10.1038/s41598-018-33392-0, published online 08 October 2018

In the original version of this Article, the author Hugo Murua Escobar was incorrectly indexed. This error has now been corrected.

